# Relationship between normal tension glaucoma and Flammer syndrome

**DOI:** 10.1007/s13167-017-0097-3

**Published:** 2017-06-02

**Authors:** Katarzyna Konieczka, Hyuk Jin Choi, Simone Koch, Franz Fankhauser, Andreas Schoetzau, Dong Myung Kim

**Affiliations:** 1 Department of Ophthalmology, University of Basel, Mittlere Strasse 91, CH-4031 Basel, Switzerland; 2 Department of Ophthalmology, Seoul National University Hospital Healthcare System Gangnam Center, Seoul, South Korea; 3 Augen Zentrum Fankhauser AG, Bern, Switzerland; 4 Department of Ophthalmology, “Victor Babes” University of Medicine and Pharmacology, Timisoara, Romania; 5 Department of Ophthalmology, Seoul National University Hospital, Seoul, South Korea

**Keywords:** Glaucomatous optic neuropathy, Normal tension glaucoma, Flammer syndrome, Primary vascular dysregulation, Ocular blood flow, Predictive, preventive, personalized medicine, Prediction of health problems

## Abstract

**Background:**

Besides intraocular pressure, vascular factors play a role in the pathogenesis of glaucomatous optic neuropathy. One of these potential vascular factors is Flammer syndrome. The purpose of the present study was to determine in a Korean population whether signs and symptoms of Flammer syndrome occur more often in normal tension glaucoma patients than in control subjects.

**Methods:**

Two hundred forty-six normal tension glaucoma patients and 1116 control subjects responded to a multiple-choice questionnaire asking about 15 signs and symptoms of Flammer syndrome.

**Results:**

Seven of the 15 signs and symptoms of Flammer syndrome (increased drug sensitivity, good smell perception, reversible skin blotches, tinnitus, long sleep onset time, tendency to perfectionism, and cold hands/feet) were significantly more often positive in normal tension glaucoma patients than in controls. Six additional signs and symptoms (migraines, low blood pressure, headaches, dizziness, increased pain sensation, and feeling cold) also occurred more often, but did not reach statistical significance. Only two items (low body weight and reduced feeling of thirst) were more frequently (not significant) positive in the controls.

**Conclusion:**

There is an association between normal tension glaucoma and Flammer syndrome. If future studies confirm this relationship, treatment of Flammer syndrome may help to prevent normal tension glaucoma or to slow down its progression.

## Introduction

Glaucomatous optic neuropathy (GON) is characterized by progressive retinal ganglion cell loss, typical tissue remodeling of the optic nerve head, and visual field defects.

Glaucoma patients, particularly those with normal tension glaucoma (NTG), have reduced ocular blood flow (OBF) [1–6]. The question arises, however, whether reduced OBF is just secondary to GON or whether there is also a primary vascular component. The presence of the primary component is supported by the facts that reduced OBF often precedes GON [2, 4], and the vascular dysfunction is not confined to the eye but can also be observed, for example, in the nailfold capillaries [7]. Blood flow reduction due to structural changes, such as atherosclerosis, seems to have less influence on the development of GON than a vascular dysregulation. Low blood pressure [8], increased response to cold [9], and increased level of the vasoconstrictor endothelin in the circulating blood [10] support the assumption of the role of vascular dysregulation. Such a dysfunction is often caused by a primary vascular dysregulation [11, 12], the core vascular component of Flammer syndrome (FS) [13]. The phenomenology of FS has been described in detail elsewhere [13–17]. The signs and symptoms of FS and the numbers for the corresponding references are listed in Table 1.

**Table 1:** The items asked in the Flammer Syndrome Questionnaire (FSQ) together with the corresponding references

Signs and symptoms of Flammer syndrome	Corresponding reference
Cold hands or/and feet	[18]
Reduced feeling of thirst	[19]
Low blood pressure	[20]
Dizziness	[21]
Increased response to certain drugs	[22]
Migraines	[23]
Headaches	[21]
Tinnitus	[24]
Low body weight	[25, 26]
Feeling cold	[27]
Long sleep onset time	[28]
Good smell perception	[29]
Increased pain sensation	[30]
Reversible skin blotches (red or white)	[11]
Tendency toward perfectionism	[11]

We investigated signs and symptoms of FS with the help of a questionnaire. The purpose of the study was to compare the self-perception of signs and symptoms of FS in NTG patients with those in control subjects.

As the frequency of FS signs and symptoms vary from country to country and region to region [31], we compared NTG patients with control subjects in the same area.

## Methods

### Participants

Two hundred and forty-six NTG patients (154 women and 92 men) filled out the questionnaire anonymously. At the same time, 1116 control subjects (567 women and 549 men) were recruited in public places and asked to fill out the questionnaire, also anonymously. Besides the presence or absence of NTG, we purposely did not use any inclusion or exclusion criteria in either study group. The study protocol was approved by the Institutional Review Board of Seoul National University Hospital Biomedical Research Institute (IRB No. H-1106-045-366). The study was designed and conducted in accordance with the tenets of the Declaration of Helsinki. All subjects completed the study without any complaints.

### Questionnaire

**Fig. 1:**
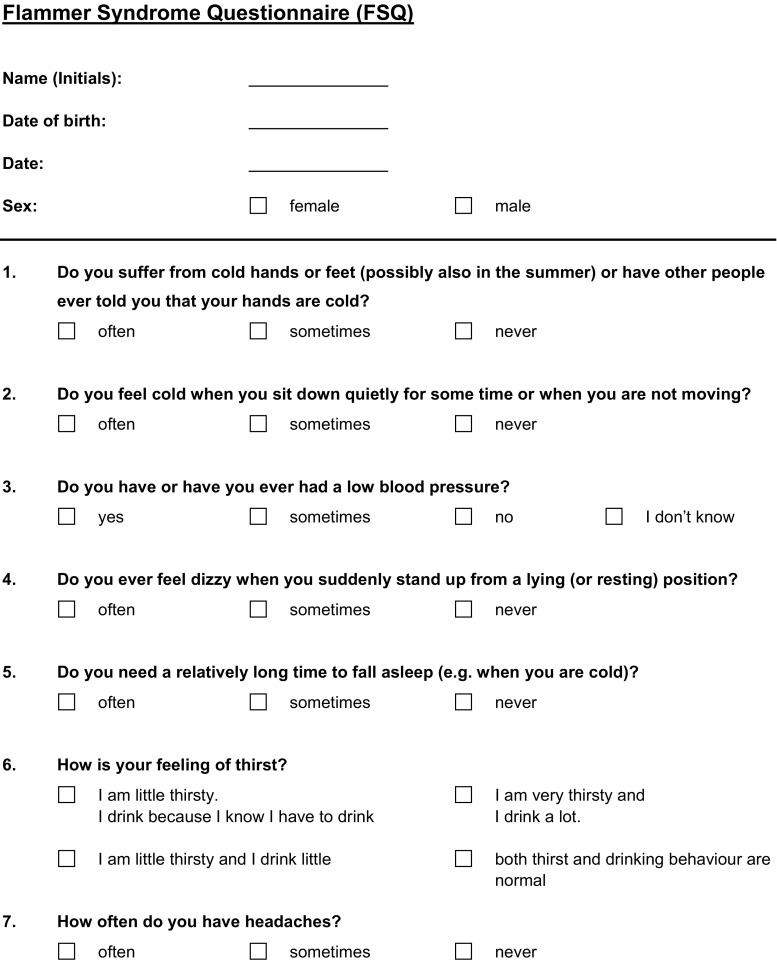

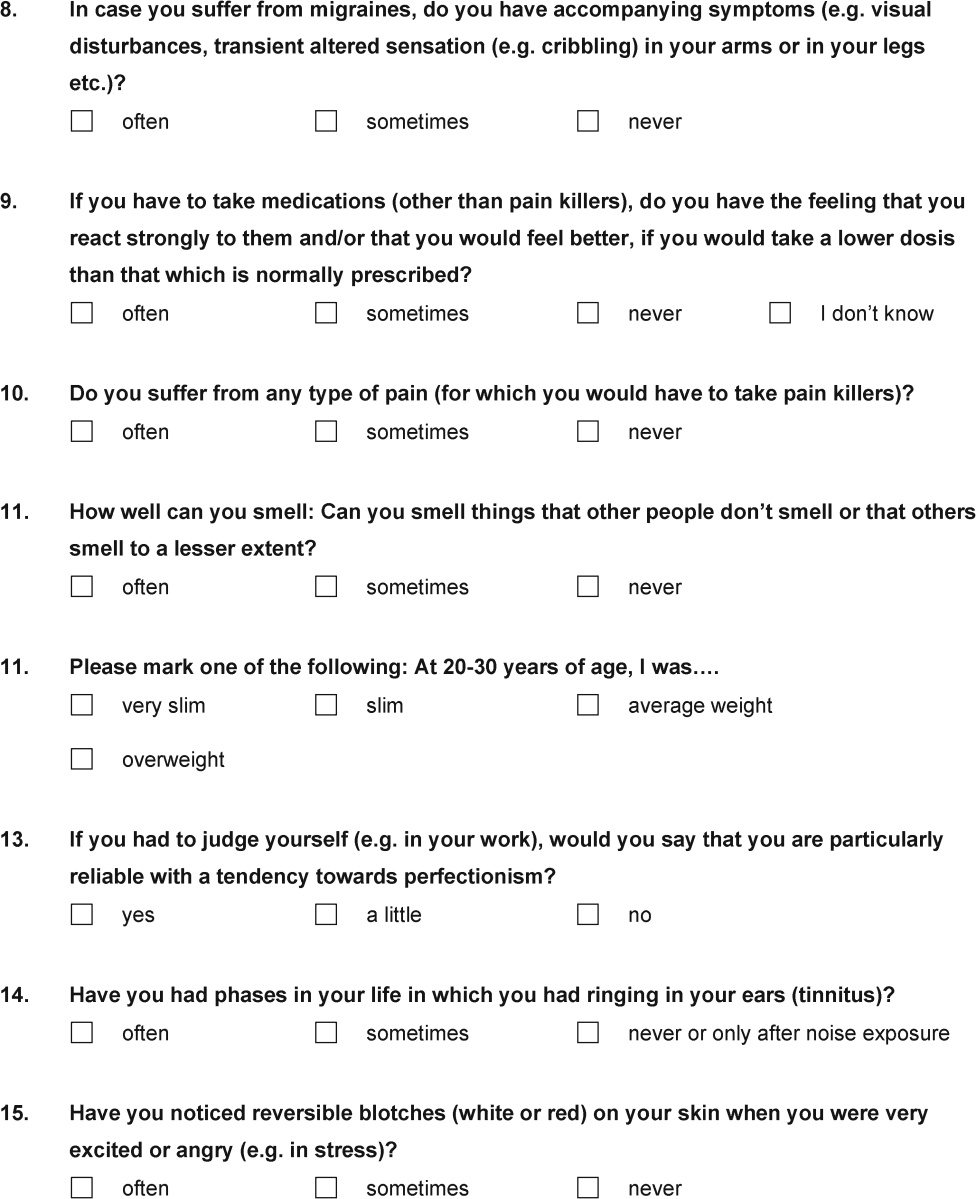
Flammer Syndrome Questionnaire (FSQ), as it was originally developed in the University of Basel, Switzerland

The Flammer Syndrome Questionnaire (FSQ), consisted of 15 items offering the following response options: «often», «sometimes», «never», or «I do not know». Table 1 lists the signs and symptoms of FS from the questionnaire with the corresponding references. The FSQ itself is depicted in Fig. 1.

### Statistical analysis

In order to study the effect of questionnaire items on NTG patients compared to control subjects, logistic regression analysis was performed, with each item as a predictor. For each item, the frequency of the most positive answer category was compared to the combined frequency of the other categories (i.e., the sum of following answer possibilities: “sometimes,” “never,” “I do not know”). Results are reported as odds ratios (ORs) and error bars expressed as ± SEM, with corresponding *p* values. Additionally, age, gender, and a possible interaction between gender and item were included in the regression models.

A *p* value of <0.05 was considered significant. This study was exploratory; therefore, *p* values were not adjusted for multiple comparisons. All analyses were done using R version 2.12.0 [32].

## Results

**Fig. 2:**
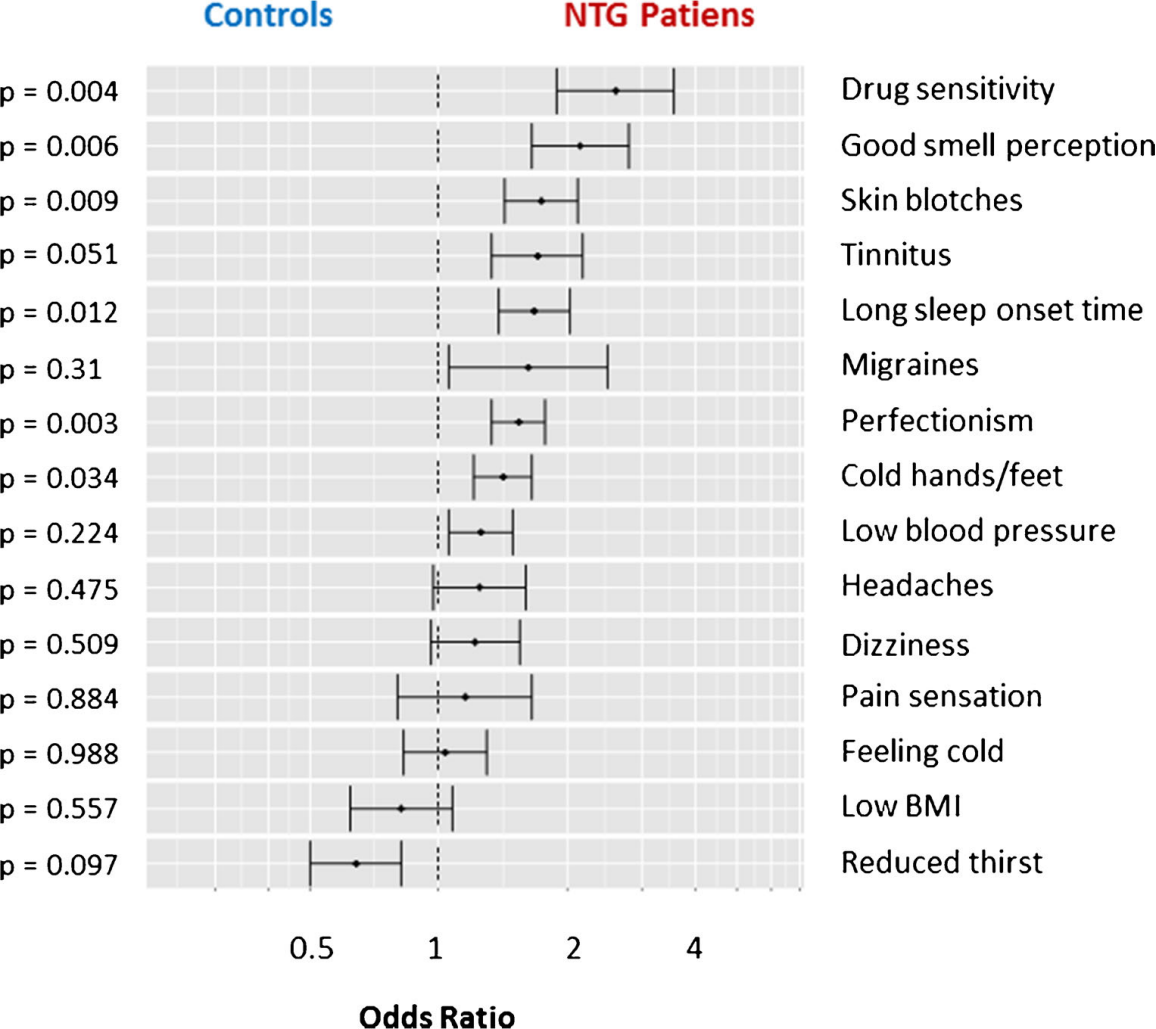
Frequency of signs and symptoms of Flammer syndrome in patients with normal tension glaucoma (NTG) (*n* = 246) in comparison to controls (*n* = 1116). For each of the questionnaire items listed in Table 1, results are presented as odds ratios and *error bars* expressed as ± SEM, with corresponding *p* values. Results are sorted by differences between the two groups, beginning with the largest one. Ratios greater than 1.0 indicate that the sign or symptom occurs more often, and ratios less than 1.0 indicate that the sign or symptom occurs less often in NTG patients than in controls

Each questionnaire item was compared between NTG patients and controls. The results are reported as ORs and sorted by differences between the two groups, beginning with the largest one (Fig. 2). Ratios greater than 1.0 indicate higher frequency of the sign or symptom in NTG patients, and ratios less than 1.0 indicate higher frequency of the sign or symptom in controls.

Seven of the 15 signs and symptoms of FS (increased drug sensitivity, good smell perception, reversible skin blotches, tinnitus, long sleep onset time, tendency to perfectionism, and cold hands/feet) were significantly more often positive in NTG patients than in controls. Six additional signs and symptoms (migraines, low blood pressure, headaches, dizziness, increased pain sensation, and feeling cold) also tended to occur more often in NTG patients (not significant), whereas two signs and symptoms (low body mass index and reduced feeling of thirst) tended to occur less often in NTG patients (not significant).

No significant interactions between gender and questionnaire items were found (*p* > 0.1); therefore, these interactions were removed from the regression models. Age and gender altered ORs only very slightly and not significantly. Therefore, ORs were not adjusted for age and gender.

**Fig. 3:**
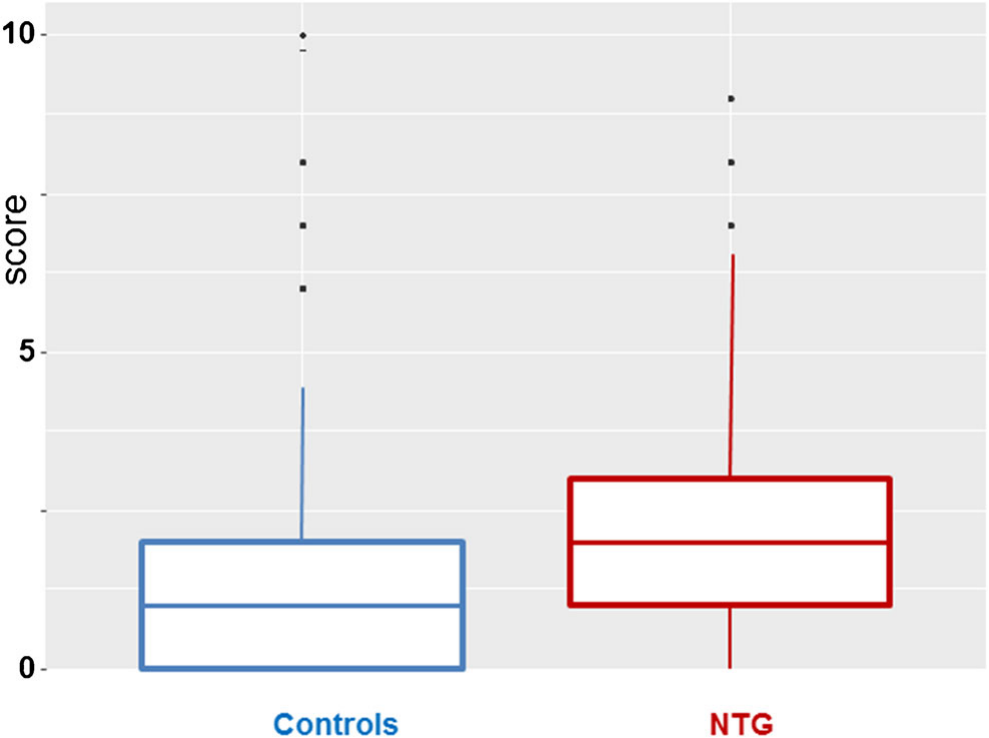
*Boxplots* representing means and distributions of the individual total scorings of Flammer syndrome (FS) in controls and NTG patients

The sum of the most positive answers was calculated for each individual. The mean and the distribution of these sums are presented as boxplots, separately for controls and NTG patients (Fig. 3). The difference between the groups was significant (*p* < 0.001) on a Wilcoxon test.

## Discussion

In the present study, the majority of FS signs and symptoms occurred more often in NTG patients than in controls.

Our results partially confirm findings already reported in the literature. Impressive and relevant is the increased drug sensitivity, a fact that has received little attention in the past. Nevertheless, increased sensitivity of NTG patients to bradykinin has already been reported [33]. Smell perception in the NTG group was significantly better than in the control group. Although this is very typical for FS [29], it is rather surprising, as smell perception drops very early in neurodegenerative diseases [34]. It has already been reported that glaucoma patients with FS have better smell perception than glaucoma patients without FS [29]. The NTG patients indicated skin blotches significantly more often than the control group. The alteration of skin perfusion is a very nice illustration of vascular dysregulation. Nevertheless, to the best of our knowledge, the literature has not yet reported this phenomenon in the context of glaucoma. Tinnitus occurred in NTG patients significantly more often, nicely demonstrating the similarity of eye and ear blood circulation. Nevertheless, this relationship has only rarely been studied well [11, 35]. Sleep onset time was prolonged in the NTG group. This is very typical for FS subjects [11] but, to the best of our knowledge, is not yet described in the context of NTG. Some studies have reported that migraines are related to NTG [36, 37]. In our NTG group, migraine occurred more often, but it did not reach statistical significance. The NTG patients considered themselves to be perfectionistic. This confirms our clinical experience, but again, to the best of our knowledge, has not yet been reported for NTG patients. Cold hands and feet, the leading symptom of FS, was indicated significantly more often in the NTG group. The observation of this symptom was the origin of the assumption of a relationship between vascular dysregulation and glaucoma [38]. Low blood pressure is part of FS and is one of the best documented risk factors for GON [39]. Low blood pressure has also been described in Korean NTG patients [40]. Nevertheless, in this study patients indicated only slightly and not significantly more often having low blood pressure than the controls. Patients obviously often do not know that they suffer from low blood pressure. This demonstrates the limits of studies based on self-perception. As the intake of antihypertensive drugs was no exclusion criterion, some patients indicating normal blood pressure may have been treated with antihypertensives.

Taken together, our results prove a relationship between FS and NTG and support the assumption that vascular dysregulation is a risk factor for NTG. The fact that non-vascular symptoms such as increased drug sensitivity or increased smell perception were also related to NTG indicates that the vascular dysregulation observed in NTG patients is often due to FS.

The involvement of FS has already been described for the following diseases: retinitis pigmentosa [11, 41–43], Leber’s hereditary optic neuropathy [44], optic nerve compartment syndrome [45, 46], retinal vein occlusions [11, 47, 48], cilioretinal artery occlusion [49], choroidal infarction [50], Susac syndrome [11], anterior ischemic optic neuropathy [11], central serous chorioretinopathy, [11], multiple sclerosis [34], and perioperative visual loss during general anesthesia [51, 52]. The relationship between FS and breast cancer has already been suggested [53] and some first results are presented in this issue of this journal. A potential, but not yet studied involvement of FS has also been discussed for the following diseases: anorexia nervosa, sudden hearing loss, ovarian cysts, and miscarriage. Future establishing of the relative risk for FS-subjects to get a FS related diseases may lead to predictive and preventive diagnostics and treatment, tailored to the person.

### Limitations of the study

This study is only based on self-perception, without any objective measurements such as quantitation of gene expression [54–57], and the results of these NTG patients cannot be extrapolated to other types of glaucoma. As all patients were from one center in South Korea, the results cannot automatically be extrapolated to other areas of the world. We did not ask for any local or systemic medication, therefore a potential influence of drugs is not excluded. There is obviously room for future studies with other designs.

## Conclusions

We provide evidence for an association between NTG and FS. It remains open at present whether treatment of FS reduces the risk for NTG and slows the progression of GON. If the relationship between NTG and FS is going to be confirmed in future studies, a tailored to the person treatment of FS may help to prevent NTG or to slow down its progression. This would be a major step toward preventive, personalized medicine.
